# Impact of Transplantation Timing on Renal Graft Survival Outcomes and Perioperative Complications

**DOI:** 10.3389/ti.2024.12202

**Published:** 2024-02-14

**Authors:** M. Uhl, T. Waeckel, E. Seizilles De Mazancourt, F. Taha, K. Kaulanjan, A. Goujon, A. Beretta, J. Papet, H. Dupuis, A. Panis, A. Peyrottes, A. Lemaire, C. Larose, L. Bettler, M. Pues, C. Joncour, G. Stempfer, T. Ghestem, P. De Sousa

**Affiliations:** ^1^ Urology, Centre Hospitalo-Universitaire Amiens Picardie, Amiens, France; ^2^ Urology, Centre Hospitalo-Universitaire Caen, Caen, France; ^3^ Urology, Centre Hospitalo-Universitaire Lyon, Lyon, France; ^4^ Urology, Centre Hospitalo-Universitaire Reims, Reims, France; ^5^ Urology, Centre Hospitalo-Universitaire Pointe A Pitre, Guadeloupe, Pointe à Pitre, France; ^6^ Urology, Centre Hospitalo-Universitaire Rennes, Rennes, France; ^7^ Urology, Centre Hospitalo-Universitaire Rouen, Rouen, France; ^8^ Urology, Centre Hospitalo-Universitaire Créteil, Paris, France; ^9^ Urology, Hôpital Européen Georges Pompidou, Paris, France; ^10^ Urology, Hôpital Saint Louis, Paris, France; ^11^ Urology, Centre Hospitalo-Universitaire Nancy, Nancy, France; ^12^ Urology, Centre Hospitalo-Universitaire Dijon, Dijon, France; ^13^ Urology, Centre Hospitalo-Universitaire Lille, Lille, France

**Keywords:** renal transplantation, graft survival, nighttime, surgery, surgical complication

## Abstract

Nighttime organ transplantation aims to decrease cold ischemia duration, yet conflicting data exists on its impact on graft function and perioperative complications. This multicenter TRANSPLANT’AFUF study including 2,854 patients, transplanted between 1 January 2011, and 31 December 2022, investigated nighttime kidney transplantation’s impact (8:00 p.m.–8:00 a.m.) versus daytime (8:00 a.m.–8:00 p.m.) on surgical complications and graft survival. Overall, 2043 patients (71.6%) underwent daytime graft, while 811 (28.4%) underwent nighttime graft. No impact was observed of timing of graft surgery on graft survival with a median survival of 98 months and 132 months for daytime and nightime grafting, respectively (*p* = 0.1749). Moreover, no impact was observed on early surgical complications (Clavien I-II = 20.95% for DG and 20.10% for NG; Clavien III-IV-V = 15.42% for DG and 12.94% for NG; *p* = 0.0889) and late complications (>30 days) (Clavien I-II = 6.80% for DG and 5.67% for NG; Clavien III-IV-V = 12.78% for DG and 12.82% for NG; *p* = 0.2444). Noteworthy, we found a significant increase in Maastricht 3 donors’ rates in nighttime transplantation (5.53% DG vs. 21.45% NG; *p* < 0.0001). In conclusion, nighttime kidney transplantation did not impact early/late surgical complications nor graft survival.

## Graphical Abstract

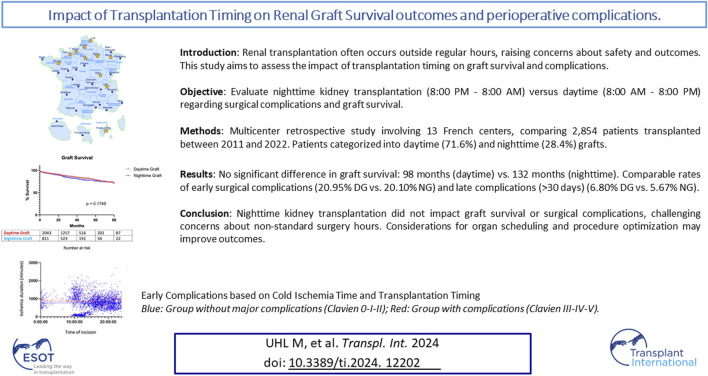

## Introduction

Renal transplantation is an unplanned activity, heavily dependent on organ retrieval and the need to minimize cold ischemia time. Consequently, it is often performed outside of regular working hours. However, the safety of surgery during non-standard hours has raised concerns over the past several years, as highlighted in various studies [1], demonstrating increased morbidity and mortality among patients operated on at night [2–6], particularly attributed to practitioner fatigue [7].

Concerning renal transplantation, fewer than ten studies have been conducted in the last two decades to evaluate the impact of nighttime interventions on short- and long-term graft functionality, as well as early and late complications [8–14]. These studies were often outdated and do not account for recent transplantation data, including the use of perfusion machines, the rise of extended criteria kidneys, and Maastricht 3 donors.

The objective of this study was to evaluate the impact of nighttime renal transplantation on graft survival and early and late surgical complications.

## Materials and Methods

### Study Design

As part of the TRANSPLANT’AFUF group led by the AFUF (Association Française des Urologues en Formation), a multicenter French retrospective database involving 13 centers was established. Inclusions were carried out successively from 2022 until 2011 for some centers. In all these healthcare facilities, kidney transplant surgeries are exclusively performed by urologists. Nevertheless, there is a lack of consensus regarding the operational protocols for managing renal transplantation.

Patients were divided into two groups based on the timing of the graft incision: daytime grafts (8:00 a.m.–8:00 p.m.) (DG) and nighttime grafts (8:00 p.m.–8:00 a.m.) (NG). 8:00 a.m. was chosen as the cut-off time because it corresponds to the change of anesthetists and OR nurses. All time points (including the moment of skin incision, the duration of the anastomosis, and the overall procedure duration) were electronically recorded during surgery and available on operative schedule. To perform a secondary analysis, a deep night graft (DNG) (11:00 p.m.–4:00) subgroup was also established.

### Parameters and Outcome Measures

Evaluated parameters included donor, graft, and recipient characteristics, graft incision and closure times, and vascular anastomosis times (time between the beginning of the first anastomosis to the end of the last anastomosis). Cold ischemia time was calculated between organ retrieval clamp and transplantation. According to the “Agence de Biomédecine” protocol, all extended criteria and Maastricht 3 kidneys must be put on hypothermic Perfusion Machine. All Maastricht 3 protocols in France are made using a normothermic extracorporeal circulation between the cardiac arrest and the retrievals of the organs [15].

The primary outcome measure was graft survival, the graft was considered non-functional in case of patient death, return to dialysis, or removal of the graft. Secondary outcome measures included graft function assessed by creatinine levels, delayed graft function (DGF, defined by the necessity of dialysis during the seven first day after transplantation), early complications (within the first postoperative month), and late complications assessed using the Clavien-Dindo classification. All these data were available on patient files.

We have distinguished between junior and senior surgeons for the analysis of complications. In our French centers, a senior surgeon has completed his or her post-internship formation.

### Statistical Analyses

Statistical analyses were performed using SPSS software. Univariate comparison was made using Chi-squared test and Fisher’s exact test for categorical variables. Continuous variables were compared using Student’s t-test or Mann–Whitney U-test (when assumptions of Gaussian distribution were not met). Graft survival was estimated using Kaplan-Meier method. A significance level of *p* < 0.05 was considered for all statistical data.

### Ethics

The study was a retrospective analysis and involved already available data on human participants and followed the 1964 Declaration of Helsinki and its later amendments. Data collection followed the French legislation concerning prospective non-interventional studies to evaluate routine care (Article Art.L1121-1-2 of French Public Health Code).

## Results

### Population Description

**FIGURE 1:**
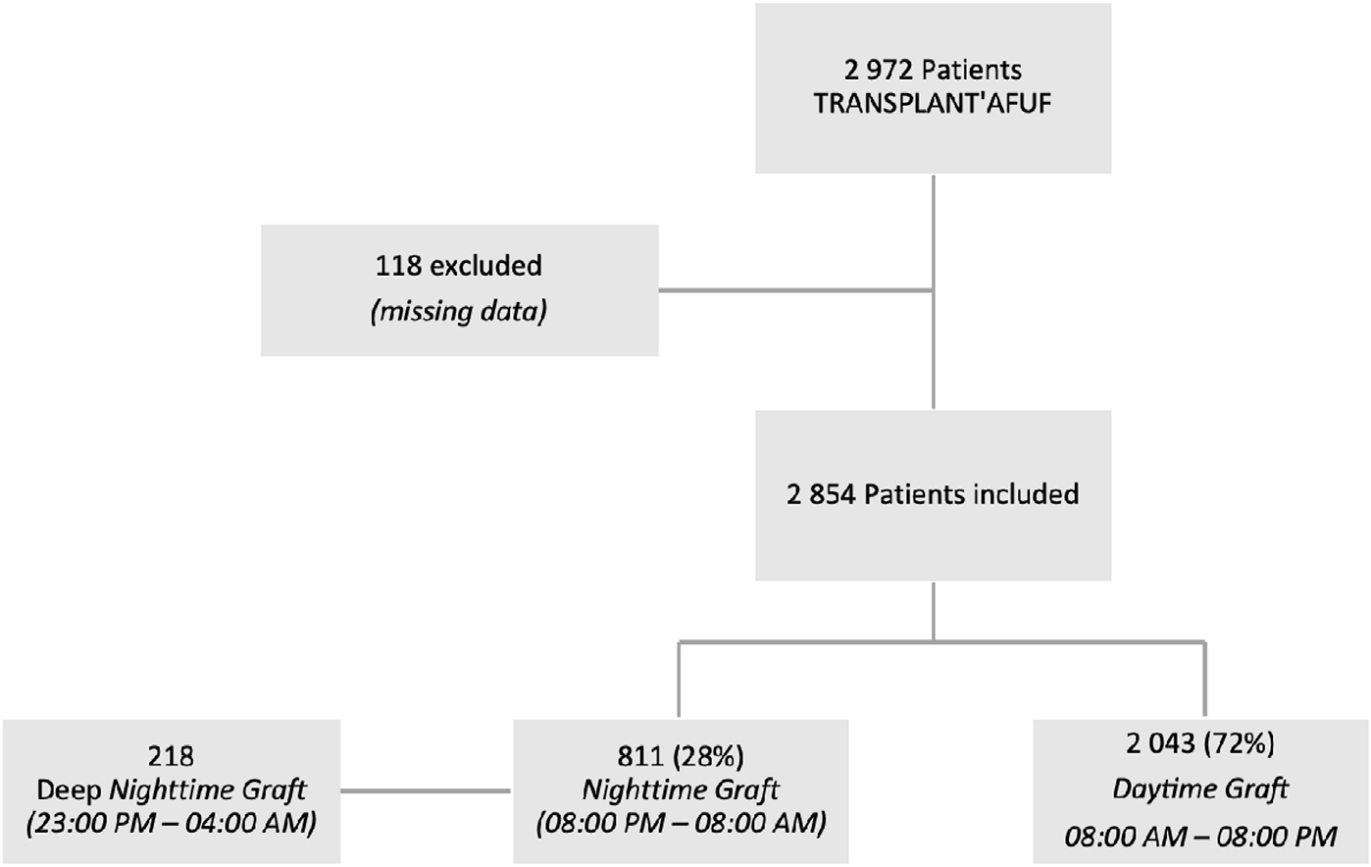
Flow chart.

Out of the 2,972 patients in the TRANSPLANT’AFUF database, 118 patients were excluded due to missing graft timing data, resulting in the inclusion of 2,854 patients between 01 January 2011 and 31 December 2022. Of these, 2,043 were daytime grafts (DG—8:00 a.m.–8:00 p.m.) and 811 were nighttime grafts (NG—8:00 p.m.–8:00 a.m.). 218 grafts were realized in deep night (DNG—11:00 p.m.–4:00 a.m.) (Figure 1).

Table 1 displays the characteristics of donors, showing significant differences in donor type with Maastricht 3 kidneys being more frequently transplanted at night (21.45% NG vs. 5.53% DG, *p* < 0.0001). Additionally, 20.80% of DG were from living donors. No differences were observed in preoperative creatinine, expanded criteria kidneys, or donor age. Regarding transplanted kidney characteristics (Table 2), NG kidneys were more often placed on machines (54.38% NG vs. 37.98% DG, *p* < 0.0001) and exhibited more arterial calcifications (27.37% NG vs. 22.47% DG, *p* < 0.017). However, no differences were found in the number of arteries or veins.

**TABLE 1:** Donor characteristics.

	Daytime graft	Nighttime graft	*p-value*
Sex (Male/Female)	1,065/978	503/308	**<0.0001** ^a^
Age (years)^b^	55 [45–56–67]	54 [47–56–65]	0.4862
Donor Type			**<0.0001** ^a^
Deceased after Brain Death (DBD)	*1,492 (73.03%)*	*619 (76.33%)*	
Maastricht Category 3 (M3)	*113 (5.53%)*	*174 (21.45%)*	
Maastricht Category 2 (M2)	*9 (0.44%)*	*10 (1.23%)*	
Living-Related Donor (LRD)	*425 (20.80%)*	*0 (0%)*	
Expanded Criteria Donors	809 (39.60%)	330 (40.69%)	0.8548
Preoperative Creatinine (µmol/L)	75 (54–86)	74 (50–86)	0.4823

^a^ Statistically significant.

^b^ Mean [first interquartile—median—third interquartile].

Italic values means number of patients.

**TABLE 2:** Renal graft characteristics.

	Daytime graft	Nighttime graft	*p-value*
Perfusion Machine	776 (37.98%)	441 (54.38%)	**<0.0001** ^a^
Multiple Arteries (>1)	481 (23.54%)	211 (26.88%)	0.1164
Arterial Calcification	459 (22.47%)	211 (27.37%)	**0.0217** ^a^
Multiple Veins (>1)	1915 (93.73%)	773 (95.31%)	0.4993

^a^ Statistically significant.

Regarding recipients, only preemptive transplantation was statistically significant (14.05% for DG vs. 6.29% for NG, *p* < 0.0001). There were no differences in terms of gender, age, BMI, cause of renal insufficiency, type of dialysis, residual diuresis, or the number of transplants. There was no difference between the mean follow up between DG and NG (30 months for DG; 28 months for NG; *p* = 0.2064) (Table 3).

**TABLE 3:** Recipient characteristics.

	Daytime graft	Nighttime graft	*p-value*
Male	1,289 (63.09%)	525 (64.73%)	0.4112
Age (years)^b^	53 [42–54–65]	54 [44–55–65]	0.3618
BMI	25.48 (22.08–28.41)	25.74 (22.65–28.69)	0.1879
Cause of ESRD			0.3296
Glomerular	*737 (36.07%)*	*309 (38.10%)*	
Vascular	*227 (11.11%)*	*96 (11.84%)*	
CTIN	*159 (7.78%)*	*56 (6.91%)*	
Polycystic	*365 (17.87%)*	*120 (14.80%)*	
Uropathy	*166 (8.13%)*	*61 (7.52%)*	
Undetermined	*214 (10.47%)*	*90 (11.10%)*	
Other	*139 (6.80%)*	*67 (8.26%)*	
Preemptive Graft	287 (14.05%)	51 (6.29%)	**<0.0001** ^a^
Dialysis Type	(*N* = 1,643)	(*N* = 731)	0.1608
Peritoneal	*199 (12.11%)*	*74 (10.12%)*	
Hemodialysis	*1,444 (87.89%)*	*657 (89.88%)*	
Residual Diuresis >50 mL/day	1,217 (59.57%)	488 (60.17%)	0.7314
≥3rd Transplant	31 (1.52%)	11 (1.36%)	0.6116
Mean follow up (months)^b^	30 [13–25–40]	28 [13–25–39]	0.2064

^a^ Statistically significant. BMI, body mass index; ESRD, end stage renal disease; CTIN, chronic tubulo-interstitial nephropathy.

^b^ Mean [first interquartile–median–third interquartile].

Italic values means number of patients.

Regarding surgery, there were more Lich-Gregoir (LG) ureteric anastomoses performed at night and more pyelo-ureteral (PU) anastomoses during the day (LG 78.56% for DG and 92.11% for NG; PU 14.93% for DG and 3.70% for NG; *p* < 0.0001). The duration of vascular anastomosis was significantly shorter during the day (50.5 min for DG vs. 55 min for NG, *p* < 0.005). The duration of cold ischemia was not statistically different between the two groups [777 min for DG (493–1,052) vs. 810 min for NG (570–1,005); *p* = 0.0541] (Table 4).

**TABLE 4:** Perioperative data.

	Daytime graft	Nighttime graft	*p-value*
Implantation Site Calcification	317 (15.52%)	112 (13.81%)	0.2783
Operation Duration (min)	180 (145–210)	178 (146–204)	0.1911
Bleeding (mL)	205 (50–300)	221 (50–300)	0.1391
Intraoperative Transfusion	98 (4.80%)	31 (3.82%)	0.3163
Senior Surgeon	829 (40.58%)	361 (44.51%)	0.0503
Urinary Anastomosis			**<0.0001** ^a^
Lich Gregoir	*1,605 (78.56%)*	*747 (92.11%)*	
Pyeloureteral	*305 (14.93%)*	*30 (3.70%)*	
Leadbetter	*12 (0.59%)*	*30 (0.37%)*	
Other	*1 (0.05%)*	*0 (0%)*	
Vascular Anastomosis Duration (min)	50.5 (35–61)	55 (39–67)	**0.0097** ^a^
Cold Ischemia Duration (min)	777 (493–1,052)	810 (570–1,005)	0.0541

^a^ Statistically significant.

Italic values means number of patients.

In a sub-group analysis, we found that, compared with DG, seniors realized significantly more DNG (40.72% DG vs. 49.08% DNG; *p* = 0.0172).

### Primary Outcome Measure

**FIGURE 2:**
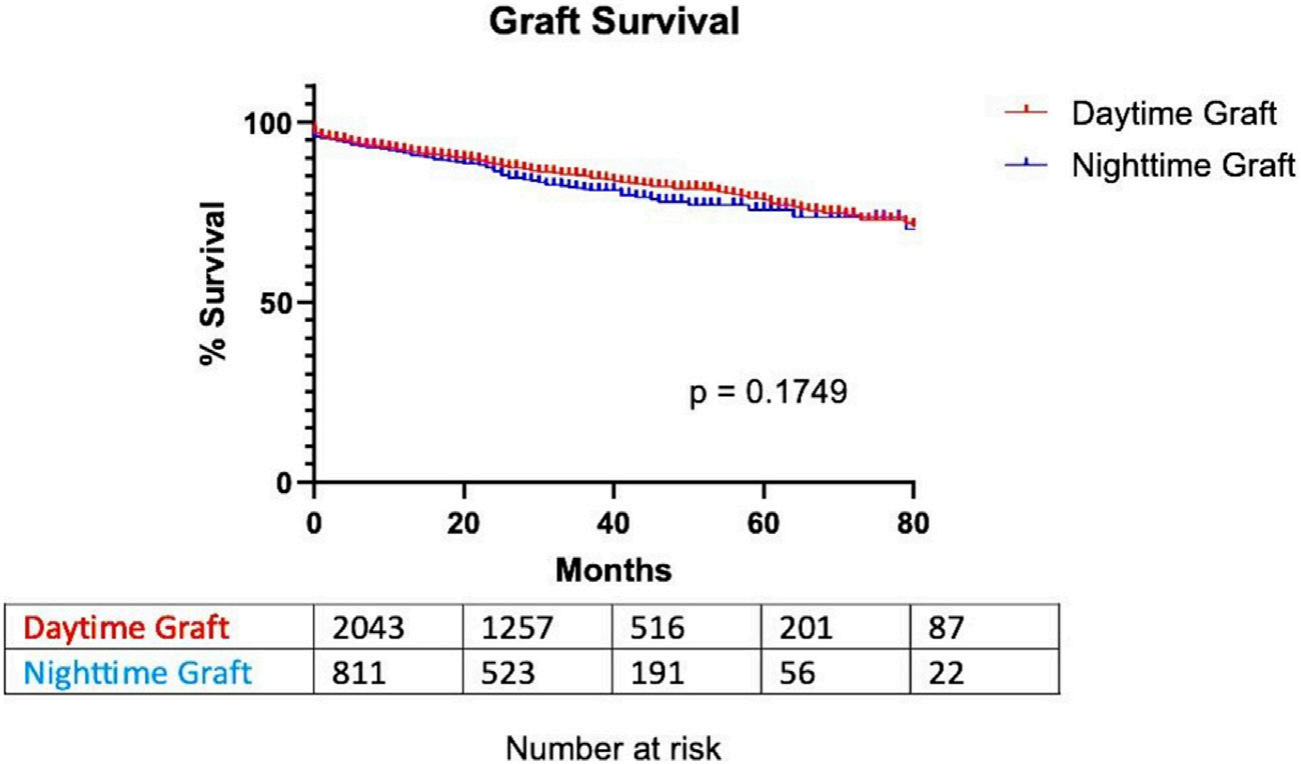
Graft survival based on transplantation timing.

Graft survival was observed, considering the graft non-functional in case of patient death, return to dialysis, or removal of the graft. There was no significant difference in graft survival between DG and NG. The time of 75% survival was 67.5 months for DG and 63.5 for NG [*p* = 0.1749; HR (day/night): 0.8695; 95% CI (0.7049; 1.073)] (Figure 2).

### Secondary Outcome Measures

Creatinine levels at 1-year post-transplant did not significantly differ between the two groups [DG: 137 μmol/L (101–157); NG: 140 μmol/L (103–158); *p* = 0.3019].

Regarding DGF, there was no statistically significant difference observed between the daytime and nighttime groups [410 DGF for DG (20.21%); 173 DGF for NG (21.54%); *p* = 0.4276] (Table 5). The occurrence of DGF was found to be correlated with prolonged anastomosis timing, irrespective of whether it occurred during the daytime (no DGF 52 min vs. DGF 64 min; *p* < 0.0001) or nighttime (no DGF 55 min vs. DGF 63 min; *p* = 0.0024).

**TABLE 5:** Postoperative data.

	Daytime graft	Nighttime graft	*p-value*
Delayed Graft Function	410 (20.21%)	173 (21.54%)	0.4276
Complication < 30 Days			0.0889
Clavien I-II	428 (20.95%)	163 (20.10%)	
* *Immediate postoperative transfusion	344 (16.84%)	134 (16.52%)	0.8388
Clavien III-IV-V	315 (15.42%)	105 (12.94%)	
* *Transplantectomy	75 (3.67%)	26 (3.21%)	
* *Thrombosis	80 (3.92%)	35 (4.32%)	
* *Surgical reintervention	241 (11.80%)	87 (10.73%)	
Urinoma	68 (3.33%)	23 (2.84%)	
Complication > 30 Days			0.2444
Clavien I-II	139 (6.80%)	46 (5.67%)	
Clavien III-IV-V	261 (12.78%)	104 (12.82%)	
* *Ureteric stenosis	69 (3.38%)	35 (4.32%)	0.2276

No significant difference was observed in surgical complications based on graft timing, either for early complications <30 days (Clavien I-II DG 20.95% vs. NG 20.10%; Clavien III-IV-V DG 15.42% vs. NG 12.94%; *p* = 0.0889) or late >30 days (Clavien I-II DG 6.80% vs. NG 5.67%; Clavien III-IV-V DG 12.78% vs. NG 12.82%; *p* = 0.2444). Looking in detail at early and late Clavien III complications (IIIa vs. IIIb), we also found no difference between DG and NG (<30 days: DG Clavien IIIa 1.57%—Clavien IIIb 9.83% vs. NG Clavien IIIa 0.97%—Clavien IIIb 8.75%; *p* = 0.4072. >30 day: DG Clavien IIIa 1.81%—Clavien IIIb 9.15% vs. NG Clavien IIIa 0.97%—Clavien IIIb 9.73%; *p* = 0.1217) (Table 5).

There was also no difference in surgical complications: immediate postoperative transfusion, graft removal, thrombosis, surgical reintervention, urinoma or ureteric stenosis (Table 5). In subgroup analysis, no significant difference was observed in surgical complications Clavien III-V for early complication <30 days (16.06% DNG vs. 12.78% DG; *p* = 0.1724).

**FIGURE 3:**
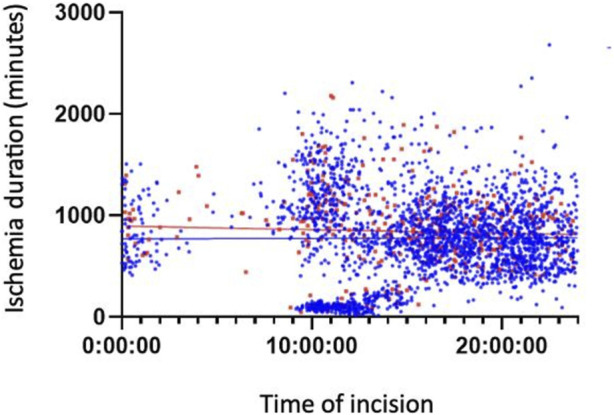
Early Complications based on cold ischemia time and transplantation timing.

**FIGURE 4:**
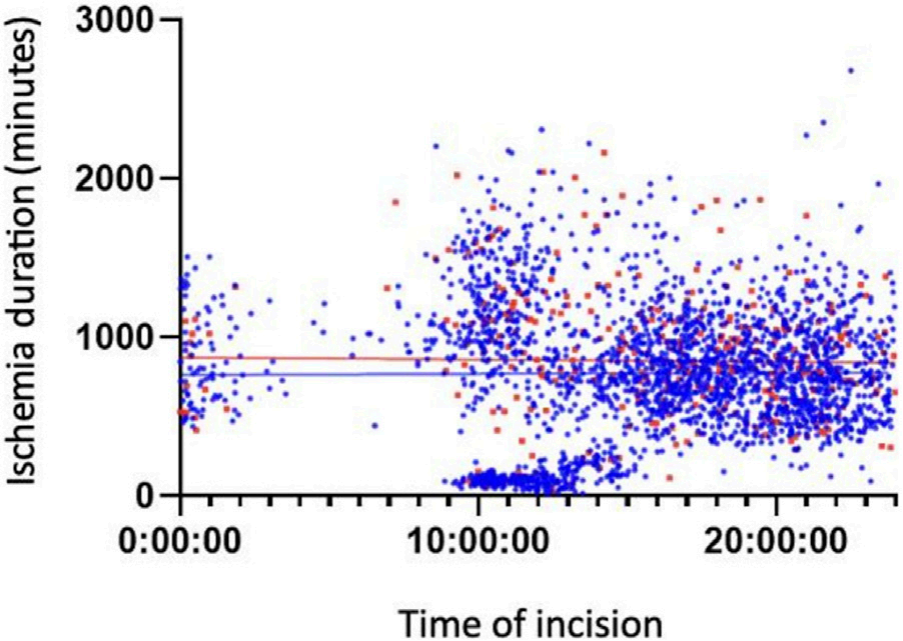
Late Complications based on Cold ischemia time and transplantation timing.

Figures 3, 4 show the association between surgical complications with cold ischemia time and transplantation timing, which revealed no difference between the two groups.

## Discussion

This French multicenter study is one of the most comprehensive analyses to date regarding the impact of transplantation timing on renal graft survival and early and late surgical complications. Organ transplantation outcomes are inconsistent, with liver grafts showing negative effects of nocturnal interventions [16], while cardiac and lung grafts remain unaffected [17]. Cold ischemia time significantly influences long-term renal graft survival [18–21]. However, surgical complications necessitating subsequent interventions have been linked to reduced graft survival [8, 22, 23], particularly vascular complications [24] and increased operative time [25]. Despite multiple retrospective analyses, these studies are often limited by small cohorts, leading to non-significant results [11–14]. Only three retrospective studies have yielded significant outcomes concerning complication rates and graft survival for nighttime kidney transplants.

Brunschot et al. [9] analyzed 4,519 kidney transplants performed between 2000 and 2013 in the Netherlands, of which 1,480 occurred at night. The results showed significantly lower technical failure rates for nighttime grafts (1%) compared to daytime grafts (2.6%). Another single-center study by Shaw TM et al. [10], involving 633 kidney transplants from 2000 to 2008, revealed increased urinary complications for grafts between 3:00 a.m. and 6:00 a.m., without a significant difference in 1-year graft survival. Fechner et al. [8] conducted a third study in 2008, comparing 260 daytime and nighttime kidney transplants between 1994 and 2004, demonstrating an elevated risk of delayed graft function recovery and vascular complications for nighttime grafts, without differences in cold ischemia time. In our study, the observed rate of surgical complications aligns with literature findings [26], showing no significant increase in surgical complications based on graft timing, despite a significant extension in vascular anastomosis time during the night.

Kidney transplants from Maastricht 3 donors were more frequently performed at night. This trend is largely attributed to the need to minimize cold ischemia time for expanded criteria grafts. However, no national protocol exists for the optimal timing of Limitation of Active Therapies (LAT) in France. The reduction of nighttime transplants could be considered by scheduling LAT for these donors earlier in the morning.

Additionally, the significant use of perfusion machines for nighttime grafts might be explained by the proportion of living-related donors (LRDs) during the day, who do not require perfusion machines, as well as the increased proportion of Maastricht 3 donors. The utilization of these machines could contribute to improved graft function [27, 28]. Similarly, the higher proportion of Maastricht 3 donors at night probably explains the gender difference, since these donors are mainly men [29].

Finally, Figures 2, 3 illustrate a cluster of morning grafts characterized by a cold ischemia window of 16–30 h, which could correspond to grafts rescheduled for the following morning to avoid procedures during the deep nighttime hours (12:00 p.m. to 08:00 a.m.). This observed behavior may be attributed to concerns regarding potential surgical complications and hesitancy within surgical teams. Our study indicates that, despite a somewhat slower pace, as evidenced by prolonged anastomosis time, potentially linked to fatigue, the initial concerns may not be substantiated. It is established that, beyond a 6-h threshold, each additional hour of cold ischemia time does impact graft survival [18–21]. Unfortunately, the limitations of our retrospective study design and the extended follow-up duration hinder our ability to conclusively demonstrate the enduring effects of cold ischemia. Facilitating easier and more direct access to the operating room holds promise for enhancing long-term graft survival, all the while maintaining organizational efficiency and ensuring the quality of work performed by surgical teams.

In conclusion, this study demonstrates that nighttime transplantations do not result in delayed graft function recovery, despite an extended vascular anastomosis time. Furthermore, these nocturnal interventions do not lead to an increased risk of early or late surgical complications. However, it’s important to note that most grafts are already scheduled for the early morning to avoid procedures during the deep nighttime period (12:00 p.m. to 08:00 a.m.), and this choice does not negatively impact graft functional recovery. However, our study could not conclude on the impact of this choice on very long-term graft survival. To reduce the number of early-night grafts, earlier LAT for Maastricht 3 donors could be considered. Additionally, the significant use of perfusion machines for nighttime grafts could contribute to the favorable graft functional recovery. We also believe that by prioritizing access to the operating room for kidney transplants, we could reduce the number of transplants delayed until the morning, and thus their cold ischemia.

## Data Availability

The raw data supporting the conclusion of this article will be made available by the authors, without undue reservation.

## Abbreviations

BMI, Body Mass Index; CTIN, chronic tubulo-interstitial nephropathy; DCD, Deceased after Cardiac Death; DG, Daytime Graft; DGF, Delayed Graft Function; ESRD, end stage renal disease; LAT, Limitation of Active Therapies; LG, Lich-Gregoir; LRD, Living-Related Donor; M2, Maastricht Category 2; M3, Maastricht Category 3; NG, Nighttime Graft; PU, pyelo-ureteral.
